# Integrated serological surveillance of acute febrile illness in the context of a lymphatic filariasis survey in Timor-Leste: a pilot study using dried blood spots

**DOI:** 10.1093/trstmh/trab164

**Published:** 2021-11-27

**Authors:** Paul Arkell, Julia Angelina, Alberina do Carmo Vieira, Johanna Wapling, Ian Marr, Merita Monteiro, Alexander Matthews, Salvador Amaral, Virginia da Conceicao, Sung Hye Kim, Daniel Bailey, Jennifer Yan, Nicholas S S Fancourt's, Susana Vaz Nery, Joshua R Francis

**Affiliations:** Menzies School of Health Research, Charles Darwin University, Darwin, NT, Australia; Imperial College London, London, UK; National Health Laboratory, Dili, Timor-Leste; National Health Laboratory, Dili, Timor-Leste; Menzies School of Health Research, Charles Darwin University, Darwin, NT, Australia; Menzies School of Health Research, Charles Darwin University, Darwin, NT, Australia; Menzies School of Health Research, Charles Darwin University, Darwin, NT, Australia; Ministry of Health, Dili, Timor-Leste; Royal Darwin Hospital, Darwin, NT, Australia; Menzies School of Health Research, Charles Darwin University, Darwin, NT, Australia; Menzies School of Health Research, Charles Darwin University, Darwin, NT, Australia; National Health Laboratory, Dili, Timor-Leste; World Health Organization, Dili, Timor-Leste; Rare and Imported Pathogens Laboratory, Porton Down, UK; Menzies School of Health Research, Charles Darwin University, Darwin, NT, Australia; Royal Darwin Hospital, Darwin, NT, Australia; Menzies School of Health Research, Charles Darwin University, Darwin, NT, Australia; Kirby Institute, University of New South Wales, Sydney, NSW, Australia; Menzies School of Health Research, Charles Darwin University, Darwin, NT, Australia; Royal Darwin Hospital, Darwin, NT, Australia

**Keywords:** dengue, dried blood spot, leptospirosis, scrub typhus, serological surveillance, Timor-Leste

## Abstract

**Background:**

Acute febrile illnesses (AFIs), including dengue, scrub typhus and leptospirosis, cause significant morbidity and mortality in Southeast Asia. Serological surveillance can be used to investigate the force and distribution of infections. Dried blood spot (DBS) samples are an attractive alternative to serum because they are easier to collect and transport and require less cold storage. We conducted a pilot study to determine the feasibility of integrating serological surveillance for dengue, scrub typhus and leptospirosis into a population-representative lymphatic filariasis seroprevalence survey in Timor-Leste using DBSs.

**Methods:**

A total of 272 DBSs were collected from healthy community participants. DBSs were analysed at the National Health Laboratory using commercially available enzyme-linked immunosorbent assays. To validate assays for DBSs, 20 anonymised serum samples of unknown serostatus were used to create dried serum spots (DSSs). These were analysed with optical densities compared with those of serum. Where low variance was observed (dengue assay) the published kit cut-offs for serum were applied to the analysis of DBSs. For the other assays (scrub typhus and leptospirosis), index values (IVs) were calculated and cut-offs were determined to be at 2 standard deviations (SDs) above the mean.

**Results:**

Of the 272 samples analysed, 19 (7.0% [95% confidence interval {CI} 4.3 to 10.7]) were positive for dengue immunoglobulin G (IgG), 11 (4.0% [95% CI 2.1 to 7.1]) were positive for scrub typhus IgG and 16 (5.9% [95% CI 3.4 to 9.4%]) were positive for leptospira IgG.

**Conclusions:**

While dengue seroprevalence was lower than in nearby countries, results represent the first evidence of scrub typhus and leptospirosis transmission in Timor-Leste. Integrated programmes of serological surveillance could greatly improve our understanding of infectious disease epidemiology in remote areas and would incur minimal additional fieldwork costs. However, when planning such studies, the choice of assays, their validation for DBSs and the laboratory infrastructure and technical expertise at the proposed location of analysis must be considered.

## Introduction

Acute febrile illnesses (AFIs) are common in the tropics and contribute significantly to morbidity and mortality among adults and children worldwide.^1,2^ In Southeast Asia, studies of febrile patients attending health facilities have identified dengue, scrub typhus and leptospirosis as major causes.^3^ These infections often present with non-specific clinical features and require laboratory confirmation for diagnosis. A lack of access to laboratory services means that treatment decisions are made empirically for many patients in remote areas.^4^ Accurate, contemporary epidemiological data on the burden and specific causes of AFIs are crucial to determine appropriate local treatment algorithms.

Timor-Leste is a Southeast Asian nation with a population of 1.2 million living in 13 municipalities. Reported cases of malaria have decreased significantly (from 37 896 in 2006 to 94 in 2016).^5^ Dengue virus is endemic and is reported through the health information system, with geographically widespread outbreaks occurring during rainy seasons since at least 2005.^6^ Otherwise, the causes of AFIs are largely unknown and it is not known whether scrub typhus or leptospirosis are endemic. Data from the 2018 National Demographic and Health Survey showed that 13% of children <5 y of age had symptoms of fever during the 2 weeks prior to participation.^7^

For many pathogens, including dengue,^8^ scrub typhus^9^ and leptospirosis,^10^ specific immunoglobulin G (IgG) antibodies can be detected in the blood for many years following infection. Determination of individual serostatus is the basis for serological surveillance, which can be used to investigate the force and distribution of infection within a population.^11^ Serial sampling can also be used to provide early warning of or aid in the investigation and characterisation of an outbreak.^12,13^ Traditionally, serological analysis is performed on serum. However, collection and transport of serum samples for surveillance can be technically challenging in remote areas. Research staff require phlebotomy skills and samples must be centrifuged and refrigerated until analysis. Furthermore, venepuncture is technically challenging in young children. Therefore many studies test routinely available samples, for example, those from blood donation or antenatal screening tests, but the generalisability of findings when these methods are used is inherently limited.^14^

Dried blood spot (DBS) samples can be collected by applying drops of whole blood, which can be obtained through finger- or heel-prick techniques, onto filter paper. Once dried, samples can be transported easily and have a lesser requirement for cold storage. DBSs have therefore been proposed as an alternative to serum for use in serological surveillance studies in remote areas.^15,16^ Potential limitations of DBS technology include variable sample volume due to incomplete saturation of the filter paper, variable performance with different filter paper varieties, the potential for sample degradation in high temperature and/or humidity conditions and the presence of inhibitors in DBS samples made from whole blood. Further research is required to validate immunoassays for DBS samples and determine the optimal methods for collection, storage and analysis across assays.^15^

Conducting community surveys for serological surveillance of infectious diseases can be time consuming and expensive and there is a need to explore the potential for their integration with other community surveys, particularly when a finger prick blood sample is being taken. Strategies for control of neglected tropical diseases, including lymphatic filariasis (LF), offer such an opportunity: LF elimination strategies rely on surveys after rounds of mass drug administration (MDA) with diethylcarbamazine (DEC) and albendazole in areas without co-endemicity of onchocerciasis and the use of rapid tests to detect LF antigen or antibody with finger prick blood.^17^ The World Health Organization (WHO) recommends integrating post-MDA surveillance as well as post-validation surveillance with other ongoing surveillance activities.^18^

We performed a pilot study to evaluate the feasibility of integrating serological surveillance of dengue, scrub typhus and leptospirosis using DBS with an LF seroprevalence survey in Timor-Leste.

## Methods

### Participants

This study took place alongside a mid-term assessment survey after the third round of MDA with DEC and albendazole for LF in Timor-Leste, which was conducted between December 2018 and February 2019. The LF survey sampled from sentinel sites (chosen based on previous national LF data) and spot-check sites (newly chosen by random sampling of clusters [hamlets] using 2015 census data) to include participants from a total of 43 clusters in 13 municipalities.^18^ In that survey, individuals >1 y of age gave a finger-prick blood sample, which was inoculated directly into two rapid diagnostic tests (RDTs) for LF *Wuchereria* antigen and *Brugia* antibody.

A pragmatic sample size of 300 was chosen for this pilot DBS study. The first 10–15 participants were recruited into the LF study from each site in seven municipalities (Bacau, Bobonaro, Cova Lima, Dili, Ermera, Liquiçá and Viqueque). There was one sentinel site each and two to three spot-check sites in each municipality, yielding 27 sites altogether. These municipalities were chosen because they were visited during the second half of the LF survey, once consumables for DBS collection were available and training of field staff had occurred. Participants’ age and gender were recorded. Prior to informed consent, participants received a verbal explanation and written information sheet. This study received ethical approval from the University of New South Wales Human Research Ethics Committee (ref: HC190056) and the Timor-Leste Human Ethics Research Committee (ref: 340/MS-INS/GDE/III/2019).

### Sample collection and handling

DBS samples were collected and handled according to a standard operating procedure (SOP) that was adapted from WHO laboratory guidance.^19^ Briefly, DBSs were created immediately after inoculation of LF RDTs using capillary blood from the same finger-prick site. Two to three drops of blood (approximate volume 100–150 μL) were placed on Whatman 903 filter paper marked with three sampling circles (12 mm diameter). After the first circle was saturated, the second then the third circles were inoculated if blood flow was sufficient. Collection cards were then dried at ambient temperature out of direct sunlight for 4 h and placed along with a desiccant sachet into a plastic zip-lock bag. The samples were transported to the National Health Laboratory (NHL) within 3 d and stored at −20°C until analysis.

### DBS elution

DBSs were eluted using a previously validated method for dengue serological analysis.^20^ Only samples with at least one fully saturated circle were included. Two 6 mm discs were cut from each collection card using a hole punch and placed into a cryotube with 250 μL of phosphate buffered saline containing 0.05% Tween. These were left overnight on an orbital shaker (120 rpm) at ambient temperature. The resulting elute was vortexed, then centrifuged for 2 min at 10 500 *g*.

### Assay validation for DBSs

The following commercial enzyme-linked immunosorbent assays (ELISAs) were used: Panbio Dengue IgG Capture ELISA: Abbott Diagnostics, 1300 E Touhy Ave, Des Plaines, IL 60018, USA, Scrub Typhus Detect IgG ELISA System: InBios International, Inc. 307 Westlake Ave. N., Suite 300, Seattle, Washington 98109 USA and SERION ELISA classic Leptospira IgG: Serion Diagnostics, Friedrich-Bergius-Ring 19, 97076 Würzburg, Germany. It was not possible to import from overseas known positive/negative serum samples to the NHL to compare and validate the ELISAs using DBS against serum samples. This was due to a lack of sample availability at collaborating reference laboratories as well as difficulties maintaining cold-chain transport to Timor-Leste during the worldwide severe acute respiratory syndrome coronavirus 2 (SARS-CoV-2) outbreak. Therefore ELISAs were evaluated for use on DBS samples in the following manner. Dried serum spots (DSSs) were created from 20 deidentified sera with unknown serostatus at the NHL. These were subsequently eluted using the above protocol and analysed. Paired DSS and serum optical density (OD) values were compared using coefficient of variance (CV) analysis.^21^ Three aliquots of each dengue and scrub typhus kit control material were also treated in the same way and included in the evaluation. This was not possible for the leptospira kit because control material is supplied prediluted. For the dengue assay, where low variance (<10) was observed, an assumption of equivalence between the two sample types was made and the published kit cut-offs for serum were applied to our analysis of DBSs. Those in the published equivocal range were assigned as positive. For the other assays (scrub typhus and leptospirosis), where high variance (>10) was observed, equivalence between the two sample types could not be assumed. Therefore sample index values (IVs) were calculated by dividing the sample OD by the mean OD of the kit positive control (in the case of the scrub typhus IgG assay) and the kit standard serum (in the case of the leptospira IgG assay). Cut-offs were determined to be at 2 standard deviations (SDs) above the mean IV of all analysed samples.

### Sample analysis

ELISAs were conducted in accordance with the manufacturer’s instructions for use (noting that none of the commercial assays were validated for DBS samples).

### Statistical analysis

Participants were grouped into children (1–15 y) and adults (>15 y). Descriptive statistics were used to compare seropositivity between age groups, gender and municipality of residence. Fisher’s exact and Mann–Whitney U tests were used to assess the significance of relationships between categorical and ordinal data, respectively, using SPSS version 26 (IBM, Armonk, NY, USA).

## Results

### Assay validation for DBSs

**Figure 1.**
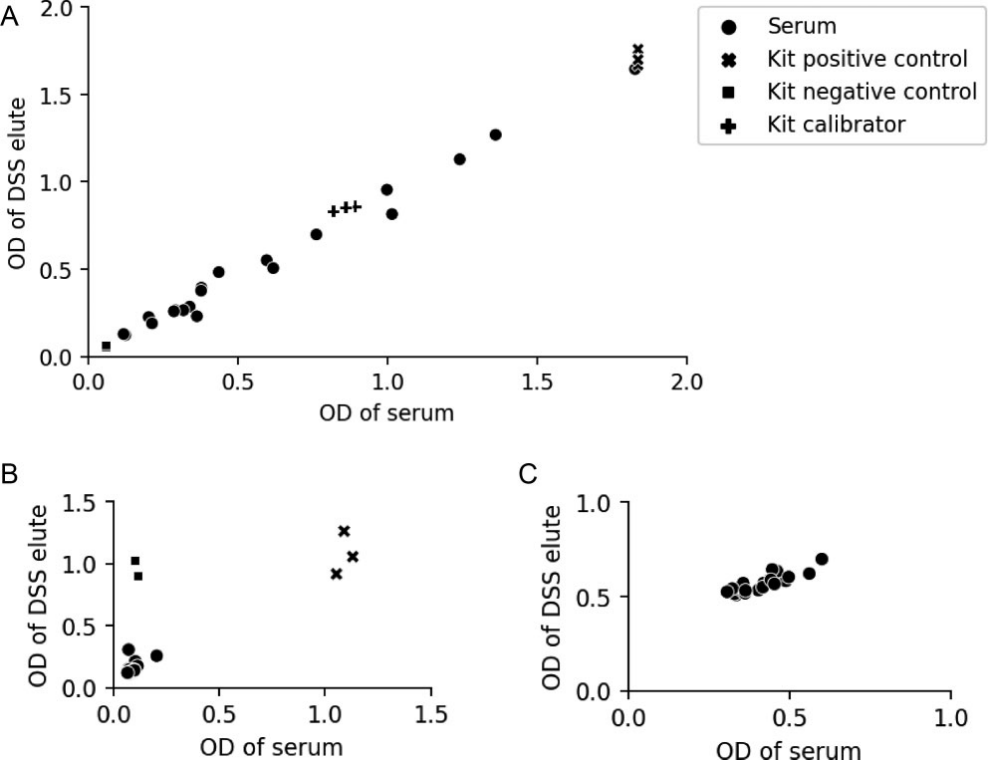
Validation of (A) dengue IgG, (B) scrub typhus IgG and (C) leptospira IgG ELISAs for DBS samples using a convenience sample of 20 stored serum samples (unknown serostatus) and aliquots of kit control materials. ODs of DSS elutes are plotted against their corresponding serum values.

When evaluating the dengue IgG ELISA by analysing a convenience sample of 20 samples with unknown serostatus and comparing the DSS OD values with those of serum, we observed low variance (CV 7.1%). This is shown in Figure 1a. The two sample types were therefore assumed to be equivalent and the published kit cut-offs for serum were applied to DBSs going forward.

For the scrub typhus and leptospira IgG ELISAs, ODs were higher from neat DSS elutes than from the serum/kit reagent counterparts. Serial dilution of elutes with kit dilution buffer reduced this and a 1:4 dilution was observed to be the best fit. Therefore DBS elutes were analysed at a dilution of 1:4. However, there remained high variance (CV for scrub typhus assay 44.3%, CV for leptospirosis assay 22.6%; see Figures 1b and 1c, respectively) and therefore kit cut-offs could not be reliably transferred to DBS results. DBS elutes were considered positive if the IV was >2 SDs above the mean IV of all analysed samples.

### Seropositivity

**Figure 2.**
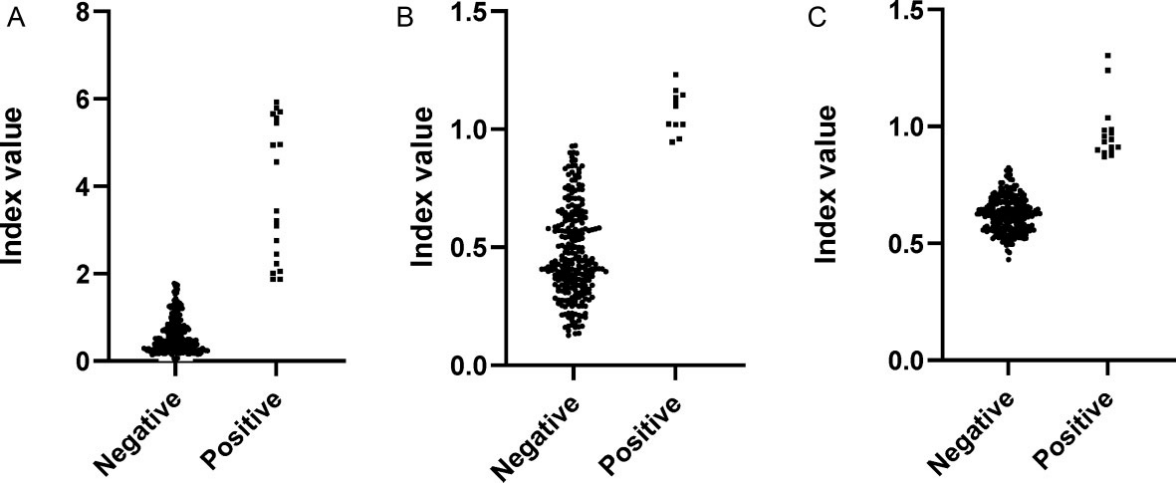
Dot plots showing IVs of individual serum samples when tested for (A) dengue IgG, (B) scrub typhus IgG and (C) leptospira IgG.

A total of 272 (88.3%) of the 308 samples received at the NHL were judged to be of sufficient quality for serological analysis. Of these, 117/264 (44.3%) were from male participants and the median age was 25 y (interquartile range [IQR] 12–43). Of the 272 samples analysed, 19 (7.0% [95% confidence interval {CI} 4.3 to 10.7%]) were positive for dengue IgG, 11 (4.0% [95% CI 2.1 to 7.1]) were positive for scrub typhus IgG and 16 (5.9% [95% CI 3.4 to 9.4]) were positive for leptospira IgG. Dengue seroprevalence appeared highest in Liquiçá (16.7%) and Bacau (12.0%) and leptospirosis seroprevalence appeared highest in Covalima (14.9%) and Bacau (10.0%). There were no other statistically significant associations between seropositivity and age, gender or municipality. Serology results are summarised in Table 1 and Figure 2.

**Table 1.** Seropositivity of dengue, scrub typhus and leptospirosis by age, gender and municipality of residence

Characteristics	Dengue IgG positive,		Scrub typhus IgG positive,		Leptospira IgG positive,		Total, n
	n (%)	Significance	n (%)	Significance	n (%)	Significance	
Gender (n=264)							
Female	12 (8.2)		7 (4.8)		9 (6.1)		147
Male	7 (5.9)	p=0.332	4 (3.4)	p=0.413	7 (5.9)	p=0.587	117
Age (years) (n=264)							
1–15	5 (6.0)		4 (4.8)		4 (4.8)		84
16–30	11 (14.1)		1 (1.3)		4 (5.1)		78
31–45	2 (4.3)		2 (4.3)		3 (6.4)		47
>45	1 (1.8)	p=0.048	4 (7.3)	p=0.361	5 (9.1)	p=0.744	55
Municipality (n=272)							
Bacau	6 (12.0)		0 (0.0)		5 (10.0)		50
Bobonaro	0 (0.0)		1 (3.4)		0 (0.0)		29
Covalima	0 (0.0)		5 (10.6)		7 (14.9)		47
Dili	3 (7.9)		2 (5.3)		1 (2.6)		38
Ermera	2 (5.9)		1 (2.9)		0 (0.0)		34
Liquiçá	7 (16.7)		0 (0.0)		0 (0.0)		42
Viqueque	1 (3.1)	p=0.015	2 (6.3)	p=0.087	3 (9.4)	p=0.007	32
Total	19 (7.0)		11 (4.0)		16 (5.9)		272

## Discussion

In this pilot study we evaluated the use of DBS samples in the serological surveillance of dengue, scrub typhus and leptospirosis in the context of a community survey to assess the status of LF in Timor-Leste. We provide the first seroprevalence data for common tropical causes of AFIs in Timor-Leste. Of the 272 samples, 19 (7.0%) were positive for dengue IgG. Dengue virus is known to be endemic in the country and is reported through the health information system, with geographically widespread outbreaks occurring during rainy seasons since at least 2005.^6^ Population-representative surveys using laboratory serological analysis of serum samples in other communities in Southeast Asian countries usually report higher dengue seroprevalence in both adults (35–91%) and children (11–60%) compared with what we found.^22–26^ This may represent a comparatively lower force of infection in Timor-Leste, which may be due to different environmental or disease-control factors. Alternatively, our study could have underestimated seroprevalence because of the small, rural sample, which may not be representative. Sample type and/or quality may have resulted in an underestimation of seroprevalence, particularly if many samples had low antibody concentrations. Similarly, the commercial dengue IgG assay we used has been optimised for diagnosis of acute dengue and differentiation of primary vs secondary infection. Therefore some samples with IgG present at low titres may have been falsely assigned as negative. This is more likely when individuals have only had one previous dengue exposure and/or when exposure occurred a long time ago. The seroprevalence of scrub typhus and leptospirosis was 4.0% and 5.9%, respectively. Both infections are endemic in many nearby Southeast Asian countries, with studies analysing serum samples from individuals in nearby countries reporting seroprevalences of 9–28%^27^ and 8–28%,^28,29^ respectively. Results from our study represent the first evidence that transmission may also occur in Timor-Leste. In this study, clinical data were not available to assess whether seropositivity related to previous exposure events or a history of a compatible illness. Serological and/or molecular testing of patients presenting with fever would be useful to further investigate the significance of these infections in Timor-Leste.

DBSs were easily collected during the LF seroprevalence survey, transported and stored. While the laboratory protocols to process and apply DBS samples to these ELISAs were relatively simple to implement, significant challenges were encountered with validation of the assays for the new sample type in Timor-Leste. In this setting it was not possible to obtain paired serum/DBS samples with known serostatus to conduct a robust validation exercise and ensure the appropriateness of published kit cut-offs. Testing a series of paired serum/DSS samples of unknown status was used as an alternative way of determining whether the assay results correlated between serum and DSS sample types. Additionally, kit positive and negative controls were included to assess correlation across the whole range of dengue IgG and scrub typhus IgG assays. This was not possible with the leptospirosis IgG assay, because kit control material is prediluted. Low variance was observed in only one of the three assays (dengue IgG). Therefore, for the remaining two assays with high variance (scrub typhus IgG and leptospirosis IgG), cut-offs were determined based on the overall distribution of the dataset, which is suboptimal. Alternative potential methods for serological cut-off determination when true seronegative/seronegative samples are not available include the following: analysis of samples from a ‘presumed unexposed’ group of individuals (i.e. those without travel to endemic areas) and setting the cut-off to be 2 or 3 SDs above the mean. We did not use this approach because such a set of DBS samples was not available in Timor-Leste; using a mixture model that assumes the presence of at least two normally distributed subpopulations within the sample population; and visually examining the plotted data set or using a ‘change point analysis’ to determine an inflection point.^30,31^ We did not use either of these statistical approaches because of this study's small sample size.

Previous studies have shown that DBS samples can be used in serological assays for dengue^20,32–35^ and scrub typhus.^36^ However, there are relatively few reports of DBS samples being used in preference to serum in programmes of active disease surveillance. This could be due to the challenges of assay validation and laboratory analysis, which may off-set any logistical benefit gained from using DBS samples. A recent review also cited requirements for assay validation against reference standards as a limitation to the use of DBS samples in the diagnosis of tropical diseases.^15^ Additionally, the opportunistic collection of DBSs during other surveys may result in suboptimal or incorrectly timed sampling for febrile illness surveillance. For example, in the present study we were unable to perform a longitudinal analysis because there were no firm plans to repeat the LF survey in Timor-Leste.

An alternative to DBS technology for serological surveillance in remote areas may be lateral flow assays (LFAs). These are cheap, portable, designed for finger-prick blood samples and have no cold storage requirements. Two recent studies have characterised the analytical performance of commercially available LFAs in detecting previous dengue infection using ELISA and virus neutralisation assays as a reference standard.^37,38^ These data may allow these to be used in serological surveillance with minimal further local validation. Furthermore, novel point-of-care assays for dengue are currently being developed for potential use in pre-vaccination (Dengvaxia) screening, which may be immediately transferrable to serological surveillance.^39^ This approach would allow the prospective integration of dengue surveillance into other epidemiological surveys. If LFAs are retained and the filter paper eluted, this could allow samples to be retested or analysed retrospectively for additional pathogens, in a fashion similar to DBSs. Novel technologies including multiplexed microsphere immunoassays may be an option for integrated serological surveillance of tropical infection. These have been used to analyse DBS samples but are currently laboratory based and require specialised equipment.^40,41^

In conclusion, this study demonstrates the feasibility of integrating the serological surveillance of causes of febrile illness into an LF seroprevalence survey using DBSs. Despite challenges relating to assay validation for the new sample type, preliminary estimates for the seroprevalence of dengue, scrub typhus and leptospirosis in Timor-Leste have been determined. This forms a basis for further investigation into the role of these conditions in causing AFIs in Timor-Leste. Further research is required to validate assays that may be used with DBSs in serological surveillance. Integrated programmes of serological surveillance have the potential to greatly improve our understanding of infectious disease epidemiology in remote areas and would incur minimal additional fieldwork costs. However, when planning such studies, consideration must be given to the choice of assay, existing validation data for DBS samples (or availability of samples for performing such a study) and the laboratory infrastructure and technical expertise at the proposed location of analysis.

## Data Availability

The data underlying this article will be shared on reasonable request to the corresponding author.
